# Novel Transfer Learning Approach for Detecting Infected and Healthy Maize Crop Using Leaf Images

**DOI:** 10.1002/fsn3.4655

**Published:** 2025-01-02

**Authors:** Muhammad Usama Tanveer, Kashif Munir, Ali Raza, Laith Abualigah, Helena Garay, Luis Eduardo Prado Gonzalez, Imran Ashraf

**Affiliations:** ^1^ Institute of Information Technology, Khwaja Fareed University of Engineering and Information Technology Rahim Yar Khan Pakistan; ^2^ Department of Software Engineering University of Lahore Lahore Pakistan; ^3^ Computer Science Department Al al‐Bayt University Mafraq Jordan; ^4^ Centre for Research Impact & Outcome Chitkara University Institute of Engineering and Technology Rajpura India; ^5^ Applied Science Research Center Applied Science Private University Amman Jordan; ^6^ Universidad Europea del Atlantico Santander Spain; ^7^ Universidade Internacional Do Cuanza Cuito Angola; ^8^ Universidad de La Romana La Romana Dominican Republic; ^9^ Universidad Internacional Iberoamericana Campeche Mexico; ^10^ Fundacion Universitaria Internacional de Colombia Bogota Colombia; ^11^ Department of Information & Communication Engineering Yeungnam University Gyeongsan‐si Korea

**Keywords:** feature extraction, plant disease detection, plant leaf detection, precision agriculture, transfer learning

## Abstract

Maize is a staple crop worldwide, essential for food security, livestock feed, and industrial uses. Its health directly impacts agricultural productivity and economic stability. Effective detection of maize crop health is crucial for preventing disease spread and ensuring high yields. This study presents VG‐GNBNet, an innovative transfer learning model that accurately detects healthy and infected maize crops through a two‐step feature extraction process. The proposed model begins by leveraging the visual geometry group (VGG‐16) network to extract initial pixel‐based spatial features from the crop images. These features are then further refined using the Gaussian Naive Bayes (GNB) model and feature decomposition‐based matrix factorization mechanism, which generates more informative features for classification purposes. This study incorporates machine learning models to ensure a comprehensive evaluation. By comparing VG‐GNBNet's performance against these models, we validate its robustness and accuracy. Integrating deep learning and machine learning techniques allows VG‐GNBNet to capitalize on the strengths of both approaches, leading to superior performance. Extensive experiments demonstrate that the proposed VG‐GNBNet+GNB model significantly outperforms other models, achieving an impressive accuracy score of 99.85%. This high accuracy highlights the model's potential for practical application in the agricultural sector, where the precise detection of crop health is crucial for effective disease management and yield optimization.

## Graphical abstract

Designed the VG‐GBNet model for feature extraction. Features involve non‐negative matrix factorization and features from the GNB model. Performance evaluation involving k‐fold and comparison with state‐of‐the‐art approaches.

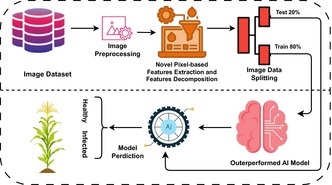

## 1 Introduction

Corn is also known as maize, and it is one of the most important crops providing solutions (Prakash and Venkataramana 2023) in many fields for humans, animals, and industries. In its traditional nutritional classification, it is the primary food for humans (Bolatova and Engindeniz 2023), which contains carbohydrates, vitamins, and minerals and is employed by most dieters in most parts of the world. Maize is commonly used in producing feeds, especially livestock feed (Karnatam et al. 2023), for poultry cattle and swine since it is regarded as a major input in this industry due to its nutritive value. Maize as an aim input is used in industry in the manufacturing of (Ranum, Peña‐Rosas, and Garcia‐Casal 2014) renewable energy‐producing commodities such as ethanol. Furthermore, it is utilized in culinary, medical, and building material industries, as well as in the creation of sweeteners, starches, and bioplastics. Maize's usefulness in so many diverse aspects of life all points to one undeniable fact (Kaushal et al. 2023). It is a staple crop that plays a vital role in the sustenance of human life as well as in animal feeds and the industrial world.

Maize is one of the leading staple crops that is consumed globally (Vanlalhruaia and Mahapatra 2023), but the crop is susceptible to several diseases that lead to reduced yields. These diseases result in dwarfism cobs with irregular and abnormal shapes and low‐quality grains, and thus, they lead to significant losses to the farmers (Vanlalhruaia and Mahapatra 2023). Sick plants may also require more inputs to combat the diseases, for instance, through buying fungicides or disease‐resistant (De Rossi et al. 2022) seeds, which will exert pressure on the available inputs. The effect of these diseases together reduces yield by approximately 10%–50% (Kumar et al. 2018) depending on the severity of the disease. Hence, disease pressures require (Blessing et al. 2022) to be controlled through integrated management, improved plant breeding, and sustainable agriculture in order to prevent yield reduction and the instability of maize yields.

In particular, there has been a significant recent development (Shoaib et al. 2023) in the use of computational methods, especially deep learning and machine learning (ML), that has led to the improvement in the early identification of diseases affecting maize crops (Ouhami et al. 2021). The precision enabled by ML and deep learning has especially proven efficient in analyzing high‐definition images acquired from maize fields (Kusumo et al. 2018). These networks can be trained to distinguish between what is normal and what is unhealthy as far as plants are concerned. They are able to diagnose problems at an early stage as compared to traditional practices (Dash, Sethy, and Behera 2023). Through incorporating these technologies with image‐based data, one is able to view large tracts of agricultural land in real time, offering farmers specific and relevant information with respect to crop status. The early identification of these diseases allows for timely measures to be taken to prevent extensive crop damage (Chu et al. 2018), thus improving production. Thus, the application of ML and deep learning in the agricultural sector makes (Taghizadeh‐Mehrjardi et al. 2020) the process of growing maize significantly more efficient and sustainable.

VGG‐16 (Etehadtavakol, Etehadtavakol, and Ng 2024) and Gaussian Naive Bayes (GNB) (Nagaraj and Kumar 2023) classifier for new transfer features were employed in extracting high‐level features from the maize crop image. The VGG‐16 architecture, a powerful deep learning model that is effective in image recognition tasks, was used to automatically identify and extract complex features (Raza et al. 2024) and patterns from the images. These high‐level features, containing basic hallmark features that characterize healthy and diseased maize, are recommended as inputs to the GNB classifier. The GNB classifier, which is famous for its simplicity as well as the ability to work with continuous data (Naseer et al. 2024), analyzed these features to classify the images and differentiate between healthy and diseased crops. Using VGG‐16 for feature extraction and GNB for classification, therefore, incorporates the benefits of both deep learning and ML, leading to a comprehensive model (Khan et al. 2023) for efficient disease detection in maize crops. The key contributions of this research are:

- For the purpose of feature learning from image data, a novel transfer learning scheme, VG‐GNBNet, is developed. For the VG‐GNBNet method, the first step involves extracting pixel‐based spatial feature data from the image data input. Thus, a novel ensemble feature set involving feature decomposition‐based matrix factorization (non‐negative) and feature vectors resulting from the GNB classifier is constructed.
- We deployed ML models using maize crop image data and the new features that have been generated. Performance comparison is done in comparison to existing works. The performance is evaluated using k‐fold cross‐validation as well. Furthermore, we determined the computational complexity of each approach.

The remaining structure of this study is described as follows: Section 2 evaluates research works on disease detection in crops, whereas Section 3 describes the recommended approach. Section 4 examines and explains the experimental outcomes. Section 5 presents the study's conclusion.

## 2 Literature Analysis

This review aims to provide comprehensive information on what is already known on the subject in an attempt to determine gaps, trends, and emerging themes that form the premise of the current study.

The conventional process of diagnosing and managing plant (Rao et al. 2022) diseases is challenging and usually requires consultation with an expert. In this regard, identification and detection systems must be more automated to be faster and more accurate on a large scale. This work also proposes a new technique known as Bi‐CNN that can be employed in diagnosing plant leaf diseases. This means fine‐tuning VGG and pruned ResNet models to act as feature extractors and joined with fully connected dense networks. With stochastic optimization, the hyperparameters aid in minimizing in fewer iterations and arrive at a more generalizable solution. In addition, the Bi‐CNN model implemented in this study can be applied to real‐life problems. After testing the model using several testing standards, the model's accuracy was found to be fairly good, with a variance of only 0. The first case showed a 27% deviation in accuracy when the test samples were multiplied fivefold. With a score of 94, the complete model met the goal since it had the highest accuracy compared to all the other models.

Various diseases that affect plant leaves can lead to plant failure and production (Sibiya and Sumbwanyambe 2019). Many diseases are known to reduce the supply of vegetables and fruits on the market and, thus, poor agricultural produce. The literature has some reports of several laboratory procedures for identifying plant leaf diseases. Diagnosing the leaf diseases with these methods was also cumbersome and confined to only a few diseases. This research uses the methodology of CNN to build a model that will be used to recognize and differentiate disease images. Neuroph used the CNN network to identify and classify malaria leaf diseases through images captured with a smartphone camera. The rational and highly effective approach to training enabled the system to be implemented almost effortlessly. This resulted in the ability of the constructed model to differentiate between three forms of maize leaf diseases and healthy maize leaves. The proposed CNN model achieved a 92% accuracy score.

The research (Fu et al. 2022) proposes a new maize spectral recovery disease identification model based on HSCNN^+^, and maize disease identification CNN decreased the cost and enhanced accuracy for actual field applications. The study also provides a spectral recovery model to estimate the HSI data from the raw maize RGB data, which is used in the disease detection network as the input data. Hence, by adding more spectral information to the raw data, the HSI reconstruction quality is satisfactorily achieved by the proposed framework. This approach greatly helps in disease recognition from the RGB images; models show good potential in disease detection. Research studies justify the usefulness of the framework in practice and prove its effectiveness in detecting infected maize at different levels of environmental interference. The CNN achieved a 96% accuracy score.

The study (Panigrahi et al. 2020) employs the supervised ML algorithms like Naive Bayes (NB), K nearest neighbor (KNN), decision tree (DT), support vector machines (SVMs), and RF for the detection of maize leaf diseases. The proposed method entails the use of image data tagged to train a classification model. RF classifier showed the best performance in disease identification when the model was tested on picture data. Diseases can be identified before they occur, and farmers can implement precautionary measures to prevent maize diseases. However, each model in the classification process consists of several constraints that may not be necessarily valid for other datasets. Higher‐dimensional datasets and different categorization algorithms can be used to implement these models.

Diseases are widely regarded as one of the biggest threats to the productivity of maize plants, with the quality and quantity of the product being severely compromised (Jasrotia et al. 2023). The use of leaves can enable the diagnosis of these diseases with ease. In this regard, this article outlines a suitable technique for diagnosing the existing diseases in the maize plant. Based on CNN, the researcher develops a customized maize plant disease identification model with input data preprocessing that involves CLAHE on each RGB channel image, performing log transform on the RGB image, and finally converting the RGB image to the HSV image. Finally, these trained models are checked out with the CNN and the SVM models that are not preprocessed. Benchmark experiments were performed on the plant village maize crop dataset to assess the efficacy of the proposed work. This proposed work affords a maximum accuracy level of 96%.

The authors developed an efficient and accurate deep CNN model in (Kasinathan and Uyyala 2023) to identify FAW insects in chief crop fields. The detection process involved a mask region–based CNN model that was trained with 798 images and tested with 57 images of Fall Armyworms (FAWs). Also, R‐CNN, Faster R‐CNN, RetinaNet, SSD, and Mask R‐CNN were used. The experimental results further revealed that Mask R‐CNN using ResNet‐101 gave the highest mAP of 94, but at a considerably lower detection time, it presented an accuracy of more than 21% for the FAW insect dataset. In particular, it took 93 s to test the proposed Mask R‐CNN. It is 6 times faster than R‐CNN and 1. It was reported 94 times faster than Faster R‐CNN. This model is essentially advantageous to farmers as it saves time, cuts costs, and has low impacts on the environment for FAW detection. In future research, further classes will be added to the FAW dataset, and CNN models will be extended by training them to use YOLO detection techniques with increased insect datasets and aerial images of crop fields taken from drones.

Plant disease scouting on large areas is expensive and fraught with inaccuracies because it is based on personal observation (Ishengoma, Rai, and Ngoga 2022). Although there are various methods of automatic identification of diseases that have proved to increase the accuracy and decrease the detection time as compared to the conventional methods, they fail to achieve the benefits of immediate detection. The current paper presents a new integrated CNN model to enhance the identification of maize leaves that have been affected by FAW. The proposed system incorporates UAV for the automatic capture of images of maize leaves, and to classify the images, a parallel CNN model with the features of both VGG16 and InceptionV3 is used. The performance of this hybrid model was compared with four existing CNN models: VGG‐16, Inception v3, Xception, and ResNet‐50. The research shows that the hybrid model is more effective and efficient than the other models, with an improvement in the training time ranging from 16% to 44% and a detection accuracy rate of 96%. It will thus bring about better and more efficient results of FAW detection in the maize crops than the current manual or the previously developed automated approaches.

In (Kang et al. 2023), the authors propose a DL model for corn leaf diseases and pest recognition with multi‐scale features and attention mechanism for three diseases and five pest detections. CBAM is incorporated into the model to improve the extraction of specific features that are pertinent to pest and disease detection. In order to improve the detection of multi‐scale objects, a multi‐feature fusion (MFF) module is incorporated into the neck network, including a weighted bidirectional feature fusion network. Outcomes learned from the experiment reveal that the model attains a figure of 85% accuracy. On the test dataset, the classification accuracy was 13%, which is 9. In other words, it achieved 59% improvements over the original CenterNet model. It is 7% less than the original CenterNet model, and it can estimate a 512 × 512 pixel image within 0.025 s, thus registering a processing speed of 23.69 frames per second. This satisfies the conditions of real‐time detection. It can be seen that the proposed model has a higher detection rate when tested under similar conditions to Faster R‐CNN, YOLOv5, SSD‐VGG, and EfficientDet‐D0 models. The created model is integrated into a web application to allow users to upload photos for fast detection and assist farmers in monitoring pest and disease occurrences.

Over the last few years, DL algorithms have proved to show high levels of accuracy in the diagnosis of crop diseases from images. The study (Haque et al. 2022) presents a new DL method to diagnose diseases in maize plants from images taken from actual fields in the ICAR‐IIMR. The focus was on four significant maize diseases: Maydis leaf blight, Turcicum leaf blight, Banded leaf, and sheath blight. Preliminary photographs, which were taken non‐intrusively, included digital cameras and smartphones with multiple backgrounds. To this end, mitigation of the class imbalance problem involved rotation and brightness enhancement of artificial images. Based on the diseased images gathered for the study, the researchers trained three different architectures using the InceptionV3 network framework with the aid of a baseline training technique. The set with the best results has an overall classification accuracy of 95%. An average precision of 99% has been achieved with an average recall of 95% and attained an impressive accuracy of 96% on a separate test dataset. Besides, it was also compared with several pre‐trained benchmark models, and it was observed that the proposed model outperformed those benchmark pre‐trained models. This shows that the adopted baseline training improves feature extraction and learning mechanisms.

Corn is one of the important food grains, and corn diseases have severe economic implications and have an impact on food security. With the use of smart devices, automatic diagnosis of corn diseases is possible, thereby reducing crop losses. The study (Mishra, Sachan, and Rajpal 2020) presents a real‐time method for identifying corn leaf diseases via a deep CNN. The results of the deep neural network are improved when hyperparameters and pooling are adjusted and optimized on a GPU system. Additional parameters were optimized to make the model more suitable for real‐time inference. A pre‐trained deep CNN model was implemented on a Raspberry Pi 3 using an Intel Movidius Neural Compute Stick, which has integrated CNN hardware blocks. While identifying the disease, the model had an accuracy of 88%. The statistic is 46%, which proves the feasibility of the concept. This corn plant disease recognition model is intended to work on smart single‐board computers such as Raspberry Pi, smartphones, and drones to demonstrate its applicability for on‐field agricultural diagnostics.

Although the above‐discussed works show promising results, the review of these works led to several limitations of these works, as given in Table 1:

- Previous researchers employed traditional approaches to identify infected corn plant parts. The employed models include traditional Ml and DL models without customization and optimization.
- Several studies utilize limited size datasets, thereby raising concerns regarding model generalizability.
- Employed DL models have higher computational complexity.
- There is a need to bring up new improved transfer learning techniques and reduce the costs of computing as well. Moreover, the performance is low.

**TABLE 1:** The literature analysis based on state‐of‐the‐art approaches.

References	Year	Technique	Dataset	Performance score (%)	Limitations
(Rao et al. 2022)	2022	Bi‐CNN	Images of plant dataset	94.84	Computational cost is increased.
(Sibiya and Sumbwanyambe 2019)	2019	CNN	Maize leaf dataset	92.0	Classical methods are used.
(Fu et al. 2022)	2022	CNN	RGB image dataset	96.0	Classical approach is used.
(Panigrahi et al. 2020)	2020	RF	Corn image dataset	91.0	Size of the dataset is low.
(Jasrotia et al. 2023)	2023	CNN and SVM	Village plant dataset	92.0	Computational cost is increased
(Kasinathan and Uyyala 2023)	2023	R‐CNN	Faw dataset	96.57	Size of dataset is low.
(Kang et al. 2023)	2023	VGG	Corn image dataset	94.4	Generalizability of dataset.
(Ishengoma, Rai, and Ngoga 2022)	2022	Xception	Faw dataset	96.0	Computational cost is increased.
(Haque et al. 2022)	2022	Inception	ICAR + IMMR dataset	96.0	Classical technique is used.
(Mishra, Sachan, and Rajpal 2020)	2020	CNN	IOT‐based dataset	88.0	Computational cost is increased.

## 3 Proposed Methodology

**FIGURE 1:**
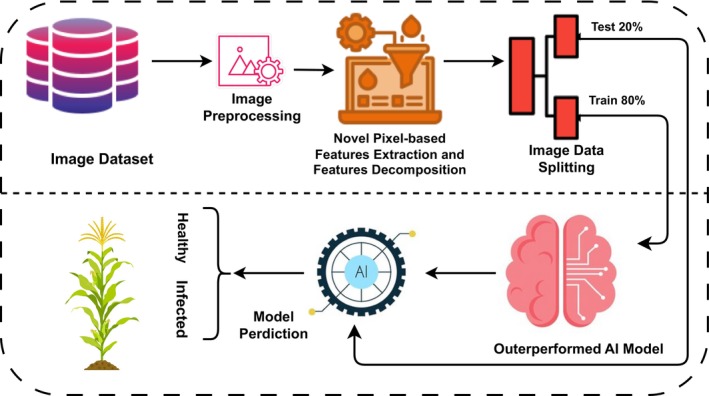
The innovative approach methodology for the detection of healthy and infected maize plants.

Figure 1 illustrates the proposed methodology of identifying healthy and infected maize crops. At first, we acquired the normal images and images of the infected ones. The provided image dataset was finally subjected to basic processes. Working with the preprocessed images resulted in a set of new features. The acquired features are split between the training and test datasets. A training data of 80% was employed to develop ML and DL models, which are described below. Several models were tested on the remaining 20% of the data.

### 3.1 Crop Leaf Image Dataset

**FIGURE 2:**
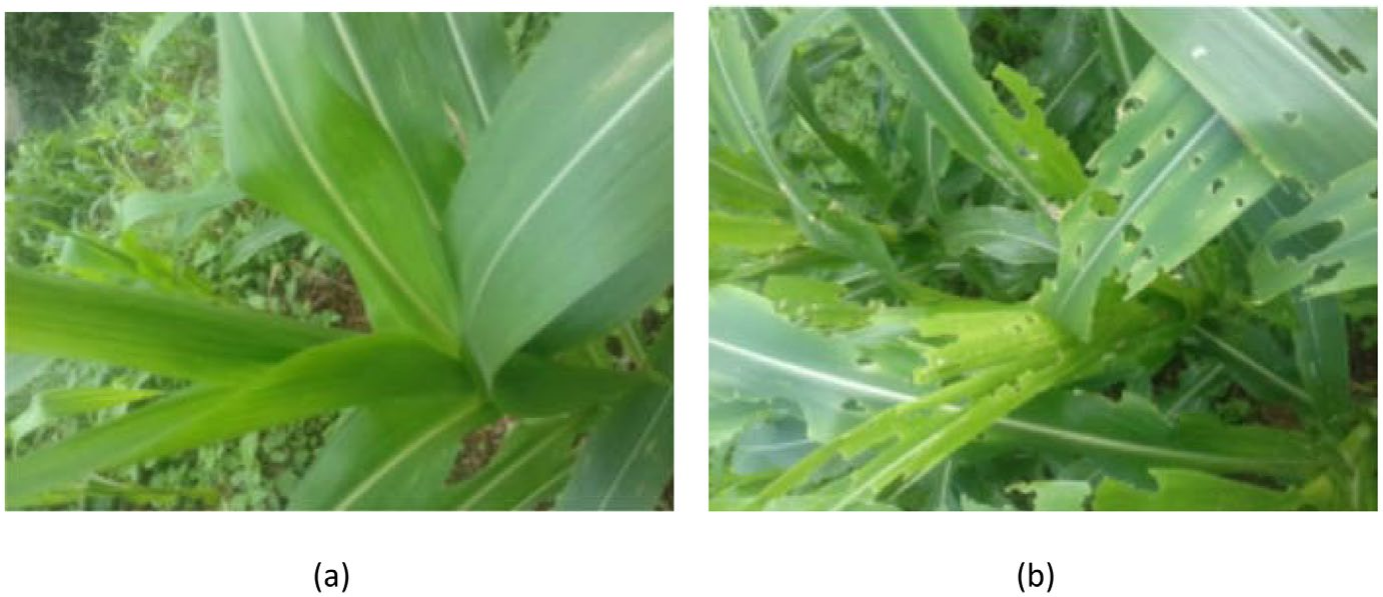
Sample images from the dataset: (a) healthy leaf and (b) infected leaf.

In this study, we utilized a maize crop image dataset. The dataset includes 4226 images of healthy and infected maize crop images (Acharya n.d.). Figure 2 shows some images from the dataset.

### 3.2 Image Processing

For better analysis, we carried out image processing steps. First, we imported maize crop image data and traversed it through the folders in the labeled directory. In the process, we endeavored to uniformly and proportionally adjust all imported images. As for the size consistency of images, the pixel data of the images were thus converted onto NumPy arrays and then changed into tensors. From the above‐mentioned processes, we gave number values for the image target labels, labeling healthy as 0 and infected as 1. When this phase was complete, we divided the dataset into testing and training sets by the distribution of 80% and 20%, respectively.

### 3.3 Novel Proposed Pixel‐Based Transfer Approach

**FIGURE 3:**
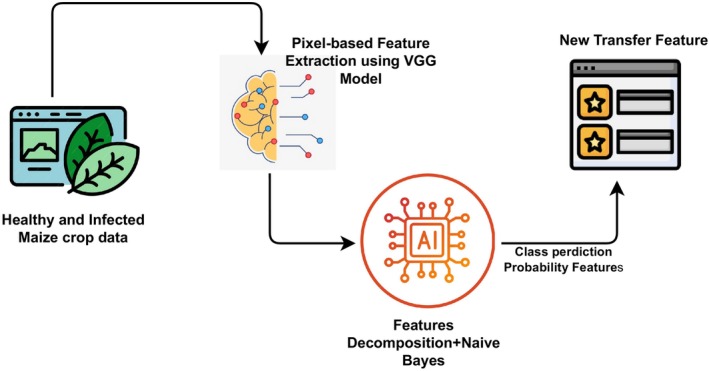
The architectural workflow of the proposed feature engineering process for healthy and infected maize crop detection.

Figure 3 represents the architectural analysis of the proposed approach in this work. In this approach to plant leaf detection, we began by extracting spatial features from leaf images using the VGG model, leveraging its convolutional layers to capture essential visual patterns and structures specific to healthy and diseased leaves. These initial features, though informative, were further transformed into a refined pixel‐based feature set using the NB model combined with a feature decomposition‐based matrix factorization (non‐negative‐NMF) mechanism. This step helped to reduce dimensionality while preserving the most relevant information for distinguishing between different plant leaf conditions. Once the new feature set was generated, we deployed various ML models to evaluate their performance on the transformed features. By testing multiple models, we aimed to identify the model that could best utilize the enhanced feature set, thus optimizing accuracy in detecting and classifying plant leaf health status. This scheme not only improved classification performance but also demonstrated the effectiveness of the feature transformation pipeline in capturing critical characteristics of plant leaves.

The proposed approach to plant leaf detection incorporates several innovative aspects that enhance both the accuracy and efficiency of disease classification. By initially using the VGG model to extract spatial features, we harnessed the power of deep convolutional networks to capture intricate patterns and structures within leaf images, which are critical for differentiating between healthy and diseased states. However, instead of relying solely on these raw deep features, we introduced a novel transformation step by applying GNB in combination with NMF. This unique pairing allowed us to refine the feature space, reducing dimensionality and highlighting the most diagnostically relevant characteristics, which are essential for plant health assessment. Moreover, the approach explores multiple ML models on the newly transformed feature set to identify the best‐performing model, thereby optimizing classification accuracy. This pipeline not only achieves high performance but also demonstrates a versatile, scalable plant leaf disease detection that can be adapted to other domains where efficient and accurate feature representation is crucial. This innovative integration of deep learning with probabilistic transformation techniques highlights a sophisticated and robust solution in the field of plant pathology. The step‐wise algorithm for the proposed model is described in Algorithm 1.

ALGORITHM 1 **Proposed pixel‐based transfer algorithm.**

**Input:** Leaf images.

**Output:** Enhanced feature set.

initiate;

1. *𝑉𝐺𝐺*_*𝑡𝑓*_ ← *𝑉𝐺𝐺*_*𝑡𝑟𝑎𝑖𝑛𝑖*_(*𝐷*_*𝑙*_) // *𝐷*_*𝑙*_ are the Pixel‐based transfer features and *𝑊*_*𝑓*_ are input leaf images.
2. *𝐹*_*𝑃𝑟𝑒𝑑*_ ← {*𝑁𝑀𝐹*_*𝐹*_ (*𝑉 𝐺𝐺*_*𝑡*_) + *𝐺𝑁𝐵*_*𝐹*_ (*𝑉 𝐺𝐺*_*𝑡𝑓*_)} // here *𝑁𝑀𝐹*_*𝐹*_ (*𝑉 𝐺𝐺*_*𝑡𝑓*_) are the features decomposition and *𝐺𝑁𝐵*_*𝐹*_ (^*𝑉 𝐺𝐺*^_*𝑡𝑓*_) are probability features set.
3. *𝐹*_*𝑡*_ are the combined Pixel‐based transfer feature set.

end;

### 3.4 Applied Learning Approaches

This study utilized ML and DL methods for analyzing the performance of the proposed transfer learning approach. We trained and tested several models such as VGG‐16 GNB, KNC, LR, and RF.

#### 3.4.1 VGG‐16

The depth and high performance of the VGG‐16 (Thakur, Sheorey, and Ojha 2023) model make it suitable for use in differentiating infected from healthy maize crops. This DL model is a 16‐layer model that utilizes CNNs to capture detailed patterns in the images of the maize crop. The process starts with the input image, referred to as X, that goes through convolution operation pooling and then passes through fully connected layers

(1) $$Y_{i,j}=f\left(\sum_{m,n}X_{i+m,j+n}\cdot W_{m,n}+b\right)$$

where *Y* matrices *𝑌*_*𝑖𝑗*_ is the output feature map, *𝑊* is the filter or kernel, *𝑏* is the bias term, and *𝑓* is the activation function.

By going through these layers, image features are captured in different hierarchical levels to make evaluations on whether the images are infected or healthy. Once trained on labeled data, the precise nature of the features is interpreted by the subsequent fully connected layers to produce a sound classification, which can assist in the management of crop diseases.

#### 3.4.2 Gaussian Naive Bayes

GNB classifier is suitable for predicting healthy and infected maize crops for its reasonable probability model (Zhang et al. 2023). This model is based on the assumption that the features of the crops are independent and identically normally distributed. In the case of a new sample, the Bayes theorem is applied to make the prediction of the class of the sample. For each feature xi of a sample, the probability density function is given by

(2) $$P\left(x_{i}|y\right)=\frac{1}{\sqrt{2\pi \sigma_{y}^{2}}}\exp\left(-\frac{{\left(x_{i}-\mu_{y}\right)}^{2}}{2\sigma_{y}^{2}}\right)$$

where *𝜇*_*𝑦*_ and *𝜎*_*𝑦*_^2^ are the mean and variance of the feature in class *𝑦*.

Calculating these probabilities for each class, the GNB assigns the sample to the class with the highest posterior probability based on the Bayes theorem. This approach together with the assumption of feature independence and data that often adhere to the normal distribution makes the classification of the crops quite efficient and accurate, thus enhancing the timely management of the crops based on observed characteristics.

#### 3.4.3 Random Forest

The Rf model is a strong learning model (Khajavi and Rastgoo 2023) that is mostly used in the classification of healthy and infected crops, in this case, maize crops. This model builds a number of DTs when training the system and combines their results to enhance the classification rate. Then, each DT is constructed with a random sample of the data and a random set of features that reduces the overfitting and increases the diversity. The output of a new sample is computed as a sum of outputs from all trees in the ensemble, often using a voting system

(3) $$\text{34𝑦̂}=\text{mode}\left\{{\text{34𝑇}}_{1}\left(\text{34𝑥}\right),{\text{34𝑇}}_{2}\left(\text{34𝑥}\right),\ldots ,{\text{34𝑇}}_{\text{34𝑛}}\left(\text{34𝑥}\right)\right\}$$

where *𝑇*_*𝑖*_(*𝑥*) represents the prediction of the *𝑖*‐th decision tree for the input sample *𝑥* and mode denotes the most frequent prediction among all trees.

This boosts the efficiency of the model in detecting diseases that affect the maize crop as well as in making improved decisions regarding agriculture.

### 3.5 K Nearest Neighbor Classifier

The KNC model can be considered an easy but effective model (Naseer et al. 2024) that can be used to classify the health status of the maize crops, either as infected or not. This non‐parametric approach involves ranking the data and selecting the K data points closest to a sample and classification based on the most frequently occurring class.

(4) $$d\left(x,x_{i}\right)=\sqrt{\sum_{j=1}^{n}{\left(x_{j}-x_{i,j}\right)}^{2}}$$

where *𝑥* is the sample to be classified, *𝑥*_*𝑖*_ is a neighboring data point, and *𝑥*_*𝑗*_ and *𝑥*_*𝑖*,*𝑗*_ are the feature values of the sample and the neighbor, respectively.

The predicted class *𝑦̂* for the sample is then determined by

(5) $$\text{34𝑦̂}=\text{mode}\left\{{\text{34𝑦}}_{\text{34𝑖}1},{\text{34𝑦}}_{\text{34𝑖}2},\ldots ,{\text{34𝑦}}_{\text{34𝑖34𝑖𝑘}}\right\}$$

where ^*𝑦*^_*𝑖*1_, ^*𝑦*^_*𝑖*2_, …, ^*𝑦*^_*𝑖𝑘*_ are the classes of the KNNs. The use of this approach facilitates the classification of new samples, which is very helpful in cases where one wants to determine the probable health state of a maize crop by evaluating on the characteristics it displays.

#### 3.5.1 Logistic Regression

The LR model is a widely used technique of statistical classification to distinguish healthy and diseased maize crops (Zhao et al. 2023). This model is expected to predict the possibility of a given sample adopting a certain class (healthy or infected) due to its features. The probability (*𝑦* = 1 ∣ *𝑥*) of a sample being classified as infected, given its features *𝑥*, is modeled as

(6) $$P\left(y=1|x\right)=\frac{1}{1+\exp^{-\left(\beta_{0}+\beta_{1}x_{1}+\beta_{2}x_{2}+\cdots +\beta_{n}x_{n}\right)}}$$

where *𝛽*_0_ is the intercept, and *𝛽*_1_, *𝛽*_2_, …, *𝛽*_𝑛_ are the coefficients for the features *𝑥*_1_, *𝑥*_2_, …, *𝑥*_𝑛_.

The logistic function provides a probability of the sample being infected, given that the linear combination of features is mapped to a value between 0 and 1. The model then classifies the sample based on this probability with the help of a threshold value that may typically equal 0.5. This method is especially useful in binary classification problems such as diagnosing the health status of the maize crop and the use of probability estimates, which could be implemented in the decision‐making processes in the agricultural sector.

### 3.6 Hyperparameter Tunning

Table 2 summarizes the hyperparameters for the ML (Datta et al. 2023) and DL models that were fine‐tuned. To further improve, we validated each strategy using k‐fold cross‐validation interactive training and testing. As presented in the research results, precise tuning of the hyperparameters to optimal reveals the results we achieved for infected and healthy crop analyses.

**TABLE 2:** This study shows hyperparameter values of applied models.

Technique	Hyperparameter
KNC	‘n_neighbour’ = 5, weights = ‘uniform’, metric = ‘minkoski’, leaf_size = 30, p = ‘2’
LR	‘copy_x’ = True fit intercept = ‘True’, Positive = ‘False’, Normalize = ‘False’
RF	max depth = 20, random_state = 0, *n* estimators = 100, criterion = ‘gini’, max features‘sqrt’.
GNB	Var‐Smoothing,1e‐9.
VGG‐16	input shape = “256,256,3”, hidden layer sizes = (100), activation = ‘sigmoid’, Optimizer = ‘Adam’, alpha = 0.0001, learning rate = ‘constant’.

## 4 Results and Discussion

In this research, we propose a novel approach toward the feature extraction for the segmentation of healthy and infected maize crops. This section discusses the proposed strategy's performance measures and compares them with contemporary strategies.

### 4.1 Experimental Setup

Table 3 shows the details of the experimental setup. In the experiment, the Google Colab is utilized as a cloud‐based platform that implements Jupyter Notebook. The performance of the ML approach is measured using several measures, among them being accuracy, F1, precision, and recall.

**TABLE 3:** The experimental setup information.

Specification	Value
Model	Dell Intel(R) CPU 3.20Ghz
Programming language	Python 3.0
CPU MHZ	3300
Cache volume	5632 KB
RAM	16 GB
CPU core	1
Address volume	64 bits virtual, 48 bits physical

### 4.2 Performance Analysis Using Spatial Features

The experiments are carried out first using the spatial features for all ML models. Results given in Table 4 indicate that the highest accuracy score of 0.80 is obtained by the RF model while other models show poor performance. The performance of RF is almost similar for healthy and infected classes, yet better for determining the healthy class. The worst performance is from the KNC model that obtains only an accuracy score of 0.58, which is lower than GBN, and LR that obtains 0.61 and 0.66 accuracy scores.

**TABLE 4:** Performance analysis using spatial features.

Model	Accuracy	Class	Precision	Recall	F1 score
KNC	0.58	Healthy	0.54	0.75	0.63
		Infected	0.67	0.43	0.53
		Average	0.61	0.58	0.57
GNB	0.61	Healthy	0.66	0.34	0.45
		Infected	0.60	0.85	0.70
		Average	0.63	0.61	0.58
LR	0.66	Healthy	0.63	0.64	0.63
		Infected	0.68	0.67	0.68
		Average	0.66	0.66	0.66
RF	0.80	Healthy	0.82	0.72	0.77
		Infected	0.78	0.87	0.82
		Average	0.80	0.80	0.80

### 4.3 Performance Analysis With Proposed Transfer Learning Approach

**FIGURE 4:**
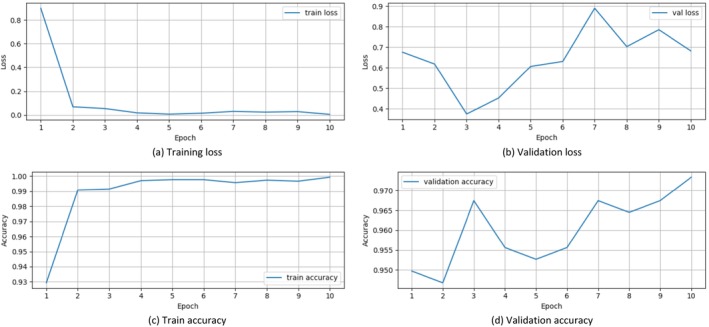
Training and validation performance of VGG‐16.

Figure 4 shows the VGG‐16 results. From the outcomes of experiments, it can be concluded that it can increase the accuracy score. The extracted spatial data features of maize crop images using VGG‐16 are as follows. Several ML algorithms used were trained on these spatial features, and the results are provided below. As for the results, it is established that the performance of the GNB approach was exceptionally high in terms of the accuracy scores. However, of all the investigated models, the only one that performed worse was the KNC model. This indicates a little improvement in the results. The performance remains below the threshold to accurately diagnose infected and healthy maize crops. Using the features obtained from VGG‐16, a minimum accuracy of 98% is obtained with all the Ml models used in this study.

Table 5 shows the results of ML models using the proposed transfer learning–based features. The outcomes of VGG‐16 suggest that it could boost classification accuracy. The features extracted from the images based on VGG‐16 lead to better training of models. The findings show that the GNB approach performed exceptionally well in comparison to other models in terms of achieving high accuracy scores with a 0.9985 accuracy score. Of all the models that were assessed, only the RF obtained lower scores with a 0.98 accuracy score. Concerning the performance, all models registered an accuracy value that ranged from 98% and above; the highest results belong to the GNB model with a 99.85% accuracy. This posits that using the suggested approach, a high accuracy can be obtained for maize crop disease detection.

**TABLE 5:** Performance analysis using the proposed transfer features.

Model	Accuracy	Class	Precision	Recall	F1 score
KNC	0.9900	Healthy	0.98	0.99	0.99
		Infected	0.99	0.98	0.98
		Average	0.99	0.99	0.99
GNB	0.9985	Healthy	0.99	1.0	0.99
		Infected	1.0	0.99	0.99
		Average	0.99	0.99	0.99
LR	0.9900	Healthy	0.99	0.97	0.98
		Infected	0.98	0.99	0.99
		Average	0.99	0.99	0.99
Rf	0.9800	Healthy	0.97	0.98	0.97
		Infected	0.97	0.97	0.96
		Average	0.98	0.98	0.98

**FIGURE 5:**
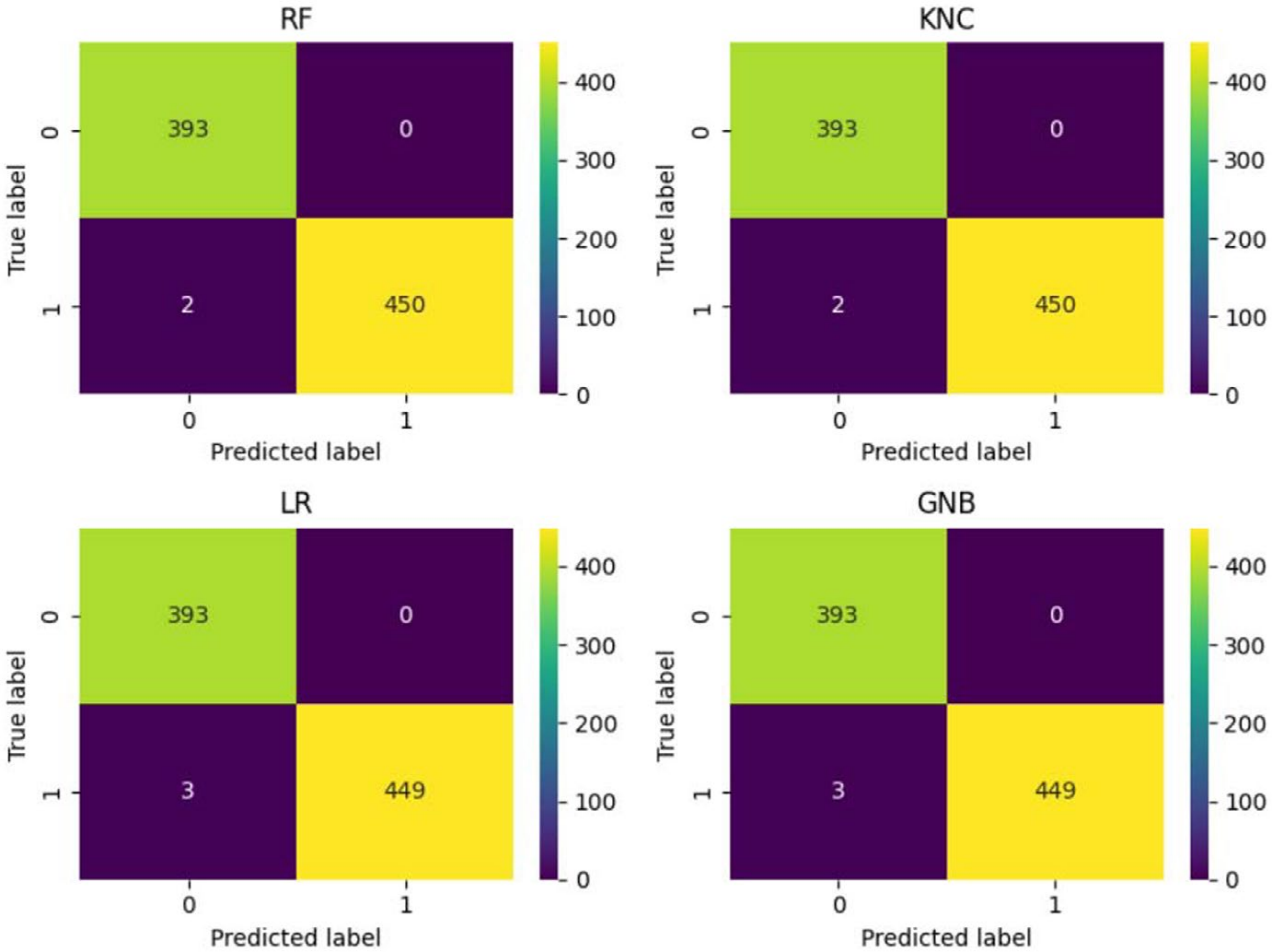
The confusion matrix for the detection of healthy and infected maize crops using the proposed approach.

Analyzing the confusion matrix of ML models in Figure 5 indicates the performance of the models concerning correct and wrong predictions. Correct predictions comprise true positives and true negatives for healthy and infected classes, respectively, while wrong predictions indicate false positives and false negatives. Results show that KNC and RF have only two wrong predictions each, while LR and GNB models make three wrong predictions each. These wrong predictions are from a total of 845 instances that were predicted by each model.

**FIGURE 6:**
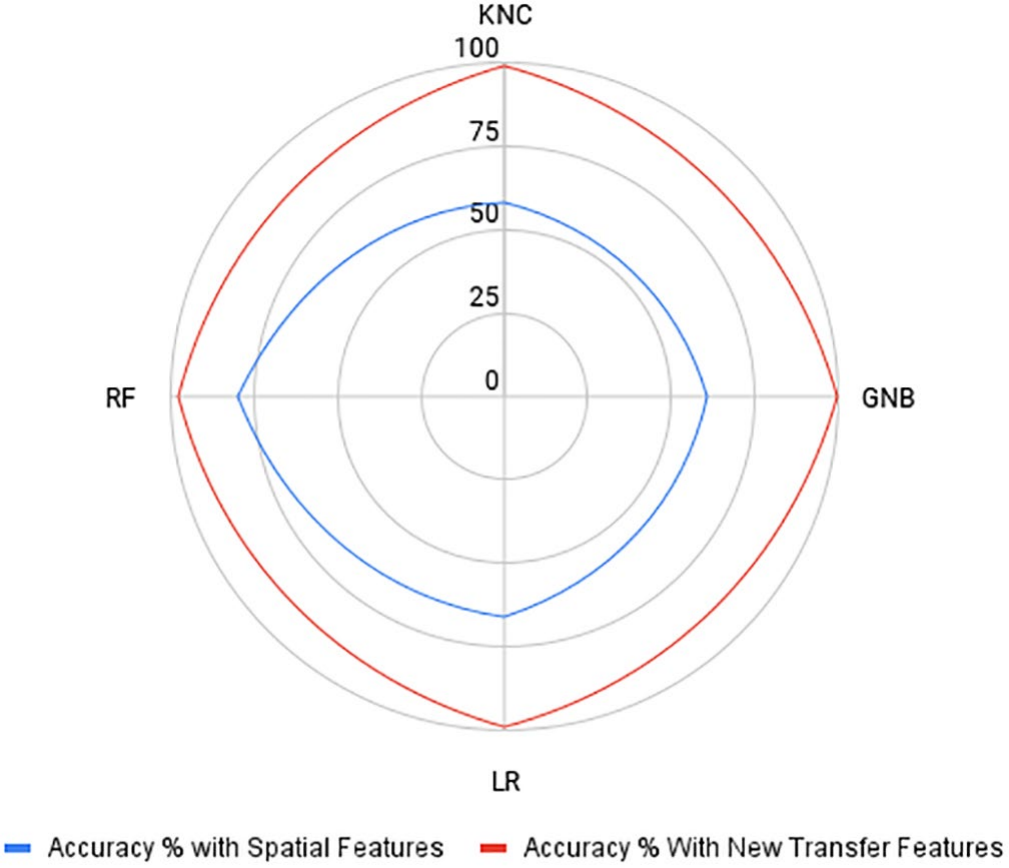
Radar chart‐based performance analysis with spatial feature and the proposed newly transfer features.

Let us compare the results of the models using spatial features and VGG‐16‐based features proposed in this study. Figure 6 compares the performance using both features. It shows that for the employed models, the proposed transfer learning features improve the performance of the models greatly. Compared to traditional spatial features, transfer learning features can better predict the maize crop disease with great accuracy.

### 4.4 K‐Fold Validation Analysis

As indicated in Table 6, we used the k‐fold cross‐validation technique with k set to 10. A well‐researched strategy would entail examining whether the Ml models in place could likely overfit the data. The validation method was carried out on all the 10 folds of the dataset. The evaluations for the model show favorable results, signifying that the proposed approach produces generalizable results and can be used for unseen data for better results. Average accuracy scores for KNC, LR, RF, and GNB are 0.96, 0.99, 0.99, and 0.9925, respectively.

**TABLE 6:** K‐fold validation analysis of the proposed approach.

Technique	K‐fold	K‐fold accuracy (%)	Standard deviation
KNC	10	0.9600	±0.0014
LR	10	0.9900	±0.0016
RF	10	0.9900	±0.0018
GNB	10	0.9925	±0.0020

### 4.5 Computational Complexity Analysis

Table 7 displays the computational complexity of the models that have been utilized in this research. The analysis implies that RF has the highest computational time of 1.2331 s. It is followed by the LR models with 0.5377 s. KNC and GNB models are favorable concerning computational complexity with 0.0324 s and 0.0145 s, respectively.

**TABLE 7:** Computational complexity analysis of the proposed approach.

Technique	Computation time
KNC	0.0324
LR	0.5377
RF	1.2331
GNB	0.0145

### 4.6 State‐of‐the‐Art Performance Comparison

Table 8 displays a comparative analysis of prior relevant state‐of‐the‐art research. Several approaches worked on crop disease detection using Ml and DL approaches, as well as a combination of both (Jasrotia et al. 2023; Kasinathan and Uyyala 2023; Haque et al. 2022; Mishra, Sachan, and Rajpal 2020). The focus of such studies is to enhance classification accuracy and improve the robustness of the model. Performance comparison shows that the proposed transfer learning–based feature engineering approach helped the models get better accuracy than existing approaches.

**TABLE 8:** The proposed model's comparison with state‐of‐the‐art approaches. Bold values shows the best performance.

References	Year	Proposed technique	Accuracy
(Jasrotia et al. 2023)	2023	CNN + SVM	92.0
(Kasinathan and Uyyala 2023)	2023	R‐CNN	96.57
(Haque et al. 2022)	2022	Inception	96.0
(Mishra, Sachan, and Rajpal 2020)	2020	CNN	85.0
Proposed	2024	VG‐GNBNet + GNB	**99.85**

## 5 Conclusion and Future Work

This research work synthesizes the use of deep learning and ML models to assess healthy and infected maize crop plants. In this study, we present VG‐GNBNet, an approach for generating pixel‐based spatial features from image data using transfer learning. VGG‐16 is used to extract features from the preprocessed data for healthy and infected maize crop leaves. It then combines the spatial features for the ensemble feature set of GNB. To analyze the performance of new features, we adopted ML models. The images considered in the study include 4225 images of healthy and infected maize crops. Compared to spatial features used for infected leaf detection, features obtained using the VGG‐16 greatly enhanced the performance of ML models. Further verification is carried out using a 10‐fold cross‐validation. An analysis of the prior work reveals that the proposed approach achieves an accuracy of 99.85%. Computational complexity analysis indicates that the proposed approach is accurate, as well as robust. In the future, we intend to develop a graphical framework that enables farmers to detect healthy and infected maize crops for precision agriculture.

## Author Contributions

**Muhammad Usama Tanveer:** conceptualization (equal), data curation (equal), writing – original draft (equal). **Kashif Munir:** conceptualization (equal), formal analysis (equal), writing – original draft (equal). **Ali Raza:** data curation (equal), formal analysis (equal), methodology (equal). **Laith Abualigah:** methodology (equal), project administration (equal), software (equal). **Helena Garay:** funding acquisition (equal), investigation (equal), visualization (equal). **Luis Eduardo Prado Gonzalez:** investigation (equal), software (equal), visualization (equal). **Imran Ashraf:** supervision (equal), validation (equal), writing – review and editing (equal).

## Conflicts of Interest

The authors declare no conflicts of interest.

## Data Availability

The data that support the findings of this study are available on request from the corresponding author.
